# Radiation Treatment Planning After Minimum Metallic Instrumentation for Patients with Spinal Metastases: A Case Series

**DOI:** 10.3390/medicina61020269

**Published:** 2025-02-05

**Authors:** Jan-Niklas Becker, Mirko Fischer, Hans Christiansen, Michael Schwake, Walter Stummer, Christian Ewelt, Niklas Benedikt Pepper, Hans Theodor Eich, Michael Müther

**Affiliations:** 1 Department of Radiation Therapy, Hannover Medical School, 30625 Hannover, Germanyfischer.mirko@mh-hannover.de (M.F.);; 2 Department of Neurosurgery, University Hospital Münster, 48149 Münster, Germany; michael.schwake@ukmuenster.de (M.S.); walter.stummer@ukmuenster.de (W.S.); 3 Department of Neurosurgery, St. Barbara-Klinik Hamm-Heessen, 59073 Hamm, Germany; cewelt@barbaraklinik.de; 4 Department of Radiation Therapy, University Hospital Münster, 48149 Münster, Germany; niklas.pepper@ukmuenster.de (N.B.P.); hans.eich@ukmuenster.de (H.T.E.)

**Keywords:** spinal tumors, radiotherapy, planning, vertebral body replacement, titanium, pedicle screws, artifacts

## Abstract

*Background and Objectives*: The utilization of non-metallic pedicle screws and rods has become a favored approach in the management of spinal tumors. An abundance of metal artifacts improves postoperative imaging and allows for precise radiation treatment planning. Under certain conditions, a vertebral body replacement (VBR) is necessary in addition to dorsal fixation. For a long time, VBR hardware was available as titanium implants only. Recently, other non-titanium products were introduced into the market. This study compares radiotherapy planning after VBR with titanium and non-titanium materials. *Materials and Methods*: This is a retrospective cohort study in a single academic center setting. VBR was performed for thoracic spinal metastatic disease. Radiation plan quality was evaluated according to the criteria of the International Commission on Radiation Units and Measurements, based on postoperative CT imaging. *Results*: Six patients with dorsal fixation and VBR were included, half of which were treated with titanium VBR and the other half with a minimum metallic implant. In addition, patients received different dorsal fixation hardware. No difference was found in terms of radiation plan quality. With non-titanium materials, visual demarcation during radiation planning was superior. *Conclusions*: This is the first study in the field to comprehensively compare radiation treatment planning after VBR using different materials. With minimum metallic implants, radiotherapy planning is equal in terms of planning but superior in terms of visual demarcation in comparison to standard titanium VBR, potentially enabling more precise radiotherapy approaches.

## 1. Introduction

Modern decision-making in the treatment of spinal metastases considers neurological compromise, radiosensitivity, spinal stability and overall patient performance. Some patients need surgical intervention for stabilization or tumor decompression [1]. Most often, stabilizing the spine can be achieved via a pedicle screw–rod construct. However, under certain circumstances, vertebral body replacement (VBR) is necessary. On a regular basis, titanium implants are used for spinal instrumentation. Such implants have the potential to induce imaging artifacts, which may subsequently compromise the accuracy of postoperative imaging and radiation treatment planning [2,3]. Therefore, minimum metallic instrumentation (MMI) has been propagated as a new standard of care for spinal tumors by various leaders in the field. With modern implants made of polyetheretherketone (PEEK) or carbon fiber-reinforced PEEK (CRF-PEEK), metal hardware components can be reduced significantly [4]. The value of CRF-PEEK dorsal instrumentation has been the subject of extensive study in recent years, including investigations into its implications for adjuvant radiation treatment planning [4,5,6,7,8,9]. Only one case series on CRF-PEEK vertebral body replacement has examined the clinical and radiological outcomes of patients with thoracolumbar tumors [10]. To date, there has been no published comparative report on the impact of minimal metallic vertebral body replacement (VBR) on radiotherapy planning for spinal metastatic disease. The objective of this study is to analyze the implications of using different VBR materials for photon radiation therapy planning in a proof-of-concept setting.

## 2. Materials and Methods

### 2.1. Study Design and Setting

This is a retrospective, uncontrolled case series and proof-of-concept study analyzing surgical cases of therapy-naïve spinal metastatic disease treated at a single academic center setting between 2012 and 2020. Postoperative imaging was used for simulated radiation treatment planning. We hypothesized that for optimal postoperative radiation treatment planning, full MMI is superior to full or partial titanium instrumentation. Data are reported respecting the updated Consensus on Preferred Reporting Of Case Series in Surgery (PROCESS) guidelines [11]. All procedures were conducted in accordance with the Declaration of Helsinki. Ethical approval for this retrospective study was obtained from the senior author’s local board (case number 2016-436-f-S).

### 2.2. Participants and Data

We searched our hospital database for patients having undergone dorsoventral instrumentation (vertebral body replacement and dorsal pedicle screw–rod fixation) using any (titanium or non-titanium) implant. To allow for better inter-individual comparability, only single-lesion spinal metastases of the thoracic spine were included. Patients without full body circumference CT appropriate for radiation planning were excluded. Neurological compromise was quantified using the Frankel Scale. Epidural Spinal Cord Compression (ESCC) was noted according to the Bilsky Scale [1]. Spinal instability was estimated using the Spine Instability Neoplastic Score (SINS) [12]. Further data points were age, sex and cancer type. The SINS, Frankel Grade, ESCC and further individual factors finally led to the decision to perform dorsoventral instrumentation complemented by tumor decompression and separation surgery, in line with current treatment paradigms [13]. After surgery, patients underwent percutaneous photon radiation therapy to optimize local tumor control. Systemic treatment was initiated after disease staging and discussion in cancer-specific tumor boards.

### 2.3. Surgical Treatment

After the induction of general anesthesia, patients were placed in the prone position. A midline skin incision was made to allow for the exposure of posterior bony structures. Pedicle screws were placed in a standard fashion using fluoroscopy and neuronavigation (Brainlab AG, Munich, Germany) in the two vertebrae above and two vertebrae below the index lesion. A laminectomy was followed by microsurgical epidural tumor resection and transpedicular tumor reduction to allow for the decompression of the spinal cord and to separate the tumor and thecal sac, as described previously [14]. Rods were placed accordingly. Autologous bone and artificial bone material (Cerasorb^®^ foam, Curasan AG, Kleinostheim, Germany) was applied to enhance on-lay posterolateral bony fusion. VBR was performed either via a dorsolateral or transthoracic approach. Procedures were executed either as a single-stage surgery or as two separate procedures, depending on individual patient and organizational factors. Implant positioning was guided by fluoroscopy and intraoperative neuronavigation. In cases of transthoracic VBR, posterior fusion was performed in a percutaneous minimally invasive fashion. According to the surgeon’s preference and the availability of specific implants, the following implants were used: Titanium VBR systems (Harms, DePuy Synthes, Raynham, MA, USA; Capri, K2M/Stryker, Kalamazoo, MI, USA), PEEK VBR system (Santorini^®^ Large, K2M/Stryker, Kalamazoo, MI, USA), CRF-PEEK screw-rod system (VADER^®^ and BlackArmor^®^, icotec AG, Altstätten, Switzerland) or a titanium screw–rod system (Everest, K2M/Stryker, Kalamazoo, MI, USA). Early postoperative CT was performed within 48 h after surgery to evaluate implant positioning. Another CT was conducted after 3 months for follow-up purposes.

### 2.4. Radiation Treatment Planning and Treatment Simulation

Based on the obtained CT scans, radiation treatment planning was performed using Monaco^®^ V5.11 software (Elekta, Stockholm, Sweden). The procedure commenced with the contouring of organs at risk, followed by the delineation of a clinical target volume (CTV) including the vertebra affected by tumor growth. A 5 mm margin was added in all directions to generate the planning target volume (PTV), accounting for inaccuracies in patient positioning and systematic variance in the linear accelerator and planning system. For each PTV, a radiation plan with 6 Mega electron Volt (MeV) photons in 10 fractions with a dosage of 3 Gray (Gy) per fraction was calculated using the Monaco Monte Carlo algorithm. The Monte Carlo algorithm in Monaco treatment planning is a highly accurate optimization method that simulates particle interactions to calculate radiation dose distributions within patient anatomy. Alternative algorithms, such as pencil beam or collapsed cone convolution, are faster but less accurate in modeling scatter and attenuation effects, especially in the presence of metallic implants. The Monte Carlo algorithm considers constraints such as tissue tolerances and tumor coverage while applying penalties to deviations from clinical objectives, ensuring precise and safe treatment planning. This approach achieves an optimal balance between delivering the prescribed dose to the target and protecting surrounding healthy tissues, which results in high conformality. Here, inverse planning was used to optimize volumetric modulated arc therapy (VMAT). VMAT is an advanced form of radiation therapy that delivers precise radiation doses to a tumor by continuously rotating the beam around the patient, allowing for greater dose control and minimum exposure to surrounding healthy tissues [15]. This optimization included the high coverage of the PTV using a high target penalty weight (100%), high conformality with a conformality function reaching a maximum of 75% of the prescribed does in a distance of 4 cm and an overall maximum dose of 109% of the prescribed dose. The treatment plan with dose distributions was finally evaluated according to the criteria of the International Commission on Radiation Units and Measurements (ICRU). From the ICRU Report 83, the following common parameters were utilized to analyze and compare plan quality [16]: D_mean_ is a dosimetric parameter in radiotherapy that represents the average dose received by the entire target volume, providing an overall assessment of the radiation dose distribution within the treated area; D_98%_ is defined as the dose received by at least 98% of the volume of interest (in this case, the PTV), representing the “near-minimum dose” as a surrogate parameter for adequate dose coverage; and D_2%_ is defined as the dose received by at most 2% of the target volume, representing the highest dose in a representative amount of tissue within the treated area (“near maximum”), helping to assess the risk of overdosage (“hot spots”). Using these parameters, the heterogeneity index (HI) was calculated for the grade of uniformity of dose distribution within the PTV. The HI can be calculated by dividing D_2%_ by the Dose to 95% (D_95%_) of the PTV:

$$\mathrm{HI}=\frac{D_{2\%}}{D_{95\%}}$$

An HI value approaching 1 indicates homogenous dose distribution within a particular volume (in this study, the PTV). Additionally, the conformity index (CI) was determined. Here, the formula represents the dividend of the volume of the PTV obtaining the prescribed dose (in this study, the prescribed dose is 30 Gy; therefore, V_prescriped dose in the PTV_ = V_30Gy_) squared and the whole volume of the PTV (V_PTV_) times the total volume in the body covered with the prescribed dose (V_irradiated with prescriped dose_):

$$\mathrm{CI}=\frac{({V\mathrm{prescriped}\;\mathrm{dose}\;\mathrm{in}\;\mathrm{the}\;\mathrm{PTV})}^{2}}{V_{\mathrm{PTV}}*V\mathrm{irradiated}\;\mathrm{with}\;\mathrm{prescriped}\;\mathrm{dose}}$$

The CI describes the volume irradiated with the prescription dose as a fraction of the target planning volume, with optimal conformity defined as a CI value approaching 1, meaning that most of the PTV is covered by the planned dose [17].

### 2.5. Statistical Analyses

Standard descriptive statistics were calculated. Continuous variables are presented as the mean with standard deviations in the dose evaluation. A two-sided non-parametric Mann–Whitney U test, also known as a Wilcoxon rank sum test, with continuity correction was performed for continuous variables. This test with continuity correction is suitable for small sample sizes, because it is a non-parametric test that does not rely on assumptions of normality. This test compares medians and ranks rather than raw data, making it robust to the effects of small sample sizes. Furthermore, continuity correction helps address the discreteness of rank sums, ensuring a more accurate *p*-value calculation in small datasets, A *p*-value below 0.05 was considered statistically noticeable within this analysis, performed with Statistical Package for Social Sciences (SPSS) version 27 (IBM, Armonk, NY, USA).

## 3. Results

### 3.1. Patient Characteristics

Over the study period, 17 patients were treated with VBR and posterior fixation for spinal metastatic disease of a single thoracic vertebra. Seven patients, for which appropriate CT data for radiation planning were available, underwent further analysis. All other patients underwent adjuvant therapies in outside institutions. The median age at surgery was 59 (50–65 IQR) years. Two patients were female. Three patients received MMI VBR; four patients were instrumented with a titanium cage. Two patients had CRF-PEEK dorsal fixation, one of which underwent full dorsoventral MMI with PEEK VBR and CRF-PEEK dorsal fixation (patient 1). See Table 1 for baseline patient characteristics. No implant-related complications were noted at the 3-month follow-up. The case of Patient 4 could not be used for further radiotherapy planning due to a cropped field of view, thus impairing accurate dose calculation.

### 3.2. Plan Quality According to Hardware Properties

Patients were stratified for VBR implant hardware properties into PEEK and titanium groups. Three patients were in each group. In each group, two titanium constructs and one CRF-PEEK dorsal fixation construct were present. Plan evaluations in terms of D_mean_, D_98%_, D_2%_, HI and CI are further shown in Table 2. Plan quality is nearly identical in terms of dosimetric parameters D_mean_, D_98%_ and D_2%_, thus dosing to 98% and 2% of the planning target volume, respectively, also comparing the mean and standard deviations. The HI and CI also gave the same quality with nearly identical standard deviation. Mann–Whitney U testing did not point out statistically noticeable differences (Table 2). Also, there was no difference between dorsal fixation systems composed of CRF-PEEK or titanium. Individual dose volume histograms (DVHs) and respective tables with DVH statistics for every case are provided in the Supplementary Materials.

For a comparison of the PEEK and titanium VBR groups, each with the mean (M) and standard deviation (SD), *p*-values are given in the last row. HI, heterogeneity index; CI, conformity index; PEEK, polyetheretherketone; VBR, vertebral body replacement; CRF-PEEK, carbon fiber-reinforced polyetheretherketone.

### 3.3. Visual Plan Comparison

**Figure 1:**
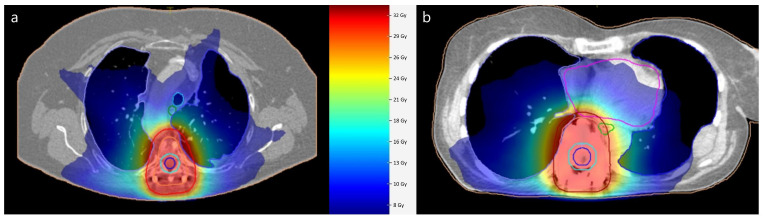
Illustration of dose distribution in volumetric modulated arc therapy radiation planning. Planning after full MMI instrumentation with PEEK VBR and CRF-PEEK dorsal fixation in patient 1 (**a**). (**b**) shows case of patient 7 after full titanium VBR and dorsal fixation. Planning target volume is outlined in red Dose is represented with color wash gradient.

Figure 1 illustrates an example of dose distributions in a patient with full MMI and full titanium instrumentation. A color wash dose distribution around the planning target volume indicates conformal distribution as used in modern radiation therapy. With PEEK VBR and CRF-PEEK dorsal fixation, the clear delineation of the vertebra, the implants and surrounding structures is noticeable. The spinal canal is especially definable. Due to the presence of metal artifacts after titanium VBR and titanium dorsal fixation, precise visual differentiation between the vertebra, implant and surrounding structures is hampered.

MMI, minimum metallic instrumentation; PEEK, polyetheretherketone; VBR, vertebral body replacement; CRF-PEEK, carbon fiber-reinforced polyetheretherketone.

## 4. Discussion

Following surgery for spinal metastatic disease, the radiation oncologist starts planning a consolidating radiation therapy. This is usually realized as fractionated photon beam irradiation via intensity modified radiation therapy (IMRT) or volumetric modulated arc therapy (VMAT). Both planning approaches are characterized by conformal dose distributions, enabling the optimal sparing of normal tissue and organs at risk with complex beam setups and dynamic collimation via multi-leaf collimators. Our case study compares radiation treatment planning after VBR with different materials. Using Monte Carlo calculations, our work shows that radiation plan quality was nearly identical in PEEK and titanium VBR systems. The standardized ICRU plan quality parameters from the ICRU Report 83 especially did not differ significantly. The same is true for the heterogeneity index and conformity index indicating similar quality in terms of heterogeneous dose distribution and target volume coverage, respectively. These results are in line with a proton/photon therapy planning study on different dorsal fixation systems composed of CRF-PEEK or titanium. Interestingly, the authors demonstrated that CRF-PEEK dorsal fixation in superior in the planning and execution of proton radiation therapy [18]. However, proton therapy is only available in highly specialized centers and is not defined as a standard of care for adjuvant radiation therapy in spinal metastatic disease.

Only a few studies have been published on non-titanium VBR. Shen et al. report on a series on an integrated custom composite polyetheretherketone/carbon fiber VBR in the treatment of bone tumors of the spine [19]. Recently, a larger series on CRF-PEEK VBR was published by Schwendner et al., reporting on a promising clinical evaluation of CRF-PEEK for vertebral body replacement in patients with thoracic and lumbar spine tumors. Still, no clinical studies have tried to objectify possible differences in treatment planning for the adjuvant photon radiation of spinal metastatic disease, a common clinical situation. Only one experimental cadaveric study reported that PEEK VBR resulted in a significantly more uniform distribution of therapeutic radiation compared with titanium [6]. There is a paucity of evidence that PEEK is inferior to titanium in spinal instrumentation in terms of mechanical properties. However, long-term arthrodesis rates following CFR-PEEK dorsal instrumentation have not yet been published in the literature [7]. Studies on other spinal implants like interbody cages and cervical anterior plating systems suggest high fusion rates [20,21].

Monte Carlo algorithms are highly advantageous for radiotherapy planning involving metal implants due to their unparalleled ability to simulate the intricate interactions of radiation with heterogeneous materials. These algorithms account for scattering, attenuation and secondary particle production, providing accurate dose calculations even in areas where simpler methods like pencil beam algorithms fall short [22]. A prerequisite for accurate photon radiotherapy is a planned CT including densitometric information. This is followed by the delineation of target volume and organs at risk, a process called contouring. Metal-related image artifacts directly find their way into radiation planning by affecting manual and semi-automatic segmentation [23]. While modern planning CT setups implement AI-based artifact suppression as well as on-board imaging during RT, this is not standard yet [24,25,26]. Additionally, the semi- or fully automated, as well as artificial intelligence-driven, segmentation of organs at risk is becoming more prevalent in modern radiotherapy planning. In this context, metal artifacts can cause errors in auto-segmentation, disrupting the workflow [23]. Therefore, PEEK will probably facilitate the transformation to emerging new artificial intelligence-driven auto-segmentations. Visually, the utilization of PEEK demonstrated an advantage in contouring paraspinal target volumes as evidenced by enhanced radiolucency and minimal artifact formation. These aspects are of particular significance to radiation oncologists. MMI can be an option for treating cases of metastases extending within and beyond the spine due to its ability to provide improved visualization, which is facilitated by a reduction in artifacts. This expands the utility of MMI in clinical practice, particularly in the context of high-precision irradiations. These techniques, also known as SBRT (Stereotactic Body Radiation Therapy), offer the advantages of delivering high-dose radiation precisely to spinal tumors while minimizing therapeutic margins and target volumes, resulting in effective tumor control with fewer treatment sessions and reduced side effects. Optimal treatment planning imaging is needed for SBRT. Here, particular parts of the vertebra are precisely segmented to only irradiate relevant volumes, as opposed to the whole vertebra in standard radiotherapy [27]. As the use of SBRT is steadily increasing, improved visualization with the precise delineation of target and surrounding structures is crucial, thus indicating the need for artifact-free implants [28]. Furthermore, the determination of the dose to the spinal cord as an organ at risk is important in spinal radiation oncology. Modern concepts also incorporate the integrated protection of the spinal cord to minimize neurotoxicity. The improved visualization after MMI enables a reasonable delineation of the spinal canal, as seen in Figure 1. Despite methodological efforts to reduce metal artifacts, MRI image quality is superior to that in MMI [4]. Especially for long-term follow-ups of benign lesions such as spinal dumbbell schwannomas in close proximity to implants, the full advantages of MMI should be exploited [29].

Although derived from only a small cohort of patients, our findings may have implications for treatment plan decisions within interdisciplinary tumor boards. There is a need for individualized interdisciplinary decision-making in the treatment of spinal metastatic disease, e.g., if there is a need for high-precision irradiations adjacent to implants. This approach underscores the importance of tailoring treatment plans to the specific needs of each patient, considering factors such as tumor location, the extent of disease and available resources. By optimizing treatment strategies in this manner, healthcare providers can achieve more cost-effective solutions without compromising patient care or outcomes. The decision to use one hardware over another can be achieved only if radiation oncologists and spine surgeons understand that with thoracic paraspinal metastatic masses, an MMI approach should be chosen.

The most immanent limitations of this case series are the small sample size, inter-individual heterogeneity and retrospective nature. Correlations between plan quality and clinical data, such as cancer type, would be of great interest and need to be addressed in future studies. It is important to note that this proof-of-concept study compares measures of treatment planning. Only comparative cadaveric analyses, such as in the pilot study performed by Jackson et al. in 2017, can contribute to demonstrating actual dose distributions [6]. Still, these experimental settings do not represent the true biology of spinal metastatic disease with varying extents of spinal and extraspinal tumor burden. The translation of the results to the treatment of lesions in other parts of the spine is desirable but poses challenges. In the cervical spine, VBR is often followed by anterior plating, which may involve titanium implants. This additionally complicates analysis and may hinder direct comparisons of thoracic spine metastatic disease, as in this study, with the rest of the spine. Despite this, it is essential to recognize that the thoracic spine is most frequently affected by osseous metastases, underlining the relevance of this study’s findings in a clinical context. The heterogeneous group of etiologies within the study population presents another limitation. The diversity of underlying causes may introduce confounding variables, making it difficult to draw meaningful comparisons in terms of oncological outcomes.

## 5. Conclusions

This is the first study in the field to approximate the impact of thoracic spine VBR hardware properties on radiation treatment planning in spinal metastatic disease. The utilization of novel minimum metallic instrumentation hardware such as PEEK does not influence the dose distribution or plan quality in photon irradiation. However, visual demarcation is notably advantageous due to the reduced number of image artifacts. This is particularly important for high-precision irradiation planning and in cases of paraspinal tumor masses.

## Figures and Tables

**Table 1:** Baseline patient characteristics.

Patient Number	Age at Surgery	Primary Cancer	Spinal Tumor Location	SINS	ESCC	Frankel Grade	VBR Implant	Dorsal Fixation Implant
1	59	Renal Cell Carcinoma	T3	10	1C	D	PEEK	CRF-PEEK
2	65	Laryngeal Cancer	T3	10	2	C	PEEK	Titanium
3	24	Malignant Peripheral Nerve Sheath Tumor	T6	7	2	E	PEEK	Titanium
4	76	Breast Cancer	T4	10	2	C	Titanium	Titanium
5	60	Renal Cell Carcinoma	T10	8	2	E	Titanium	CRF-PEEK
6	55	Malignant Mixed Müllerian Tumor	T7	8	3	D	Titanium	Titanium
7	50	NSCLC	T8	7	2	E	Titanium	Titanium

**Table 2:** Dose evaluation parameters and HI and CI for each patient.

Patient Number	Spinal Tumor Location	VBR Implant	Dorsal Fixation Implant	D_mean_	D_98%_	D_2%_	HI	CI
1	T3	PEEK	CRF-PEEK	30	28.9	30.8	0.95	1.07
2	T3	PEEK	Titanium	30	28.8	31.1	0.93	1.07
3	T6	PEEK	Titanium	30	28.6	31.5	0.88	1.09
M (SD)				30 (0)	28.8 (0.12)	31.1 (0.28)	0.92 (0.03)	1.07 (0.01)

5	T10	Titanium	CRF-PEEK	30	28.3	31.2	0.93	1.08
6	T7	Titanium	Titanium	30	28.4	31.2	0.94	1.08
7	T8	Titanium	Titanium	30	28.6	31.3	0.88	1.08
M (SD)				30 (0)	28.4 (0.12)	31.2 (0.05)	0.92 (0.03)	1.08 (0)
					
*p*-Value (Comparing PEEK and Titanium VBR)				0.99	0.12	0.66	0.99	0.64

## Data Availability

The original contributions presented in this study are included in the article. Further inquiries can be directed to the corresponding author.

## Supplementary Materials

The following supporting information can be downloaded at https://www.mdpi.com/article/10.3390/medicina61020269/s1, individual dose volume histograms (DVHs) and respective tables with DVH statistics for every planed case.

## Abbreviations

DVH	Dose volume histogram
CI	Conformity index
CTV	Clinical target volume
CRF-PEEK	Carbon fiber-reinforced PEEK
ESCC	Epidural Spinal Cord Compression
HI	Heterogeneity index
IMRT	Intensity modified radiation therapy
MMI	Minimum metallic instrumentation
PEEK	Polyetheretherketone
PTV	Planning target volume
SBRT	Stereotactic body radiation therapy
VBR	Vertebral body replacement
VMAT	Volumetric modulated arc therapy
