# Supplementary material for: Radiation Treatment Planning After Minimum Metallic Instrumentation for Patients with Spinal Metastases: A Case Series
Source: Medicina (Kaunas). 2025 Feb 5;61(2):269. doi: 10.3390/medicina61020269 (PMC11857767; doi:10.3390/medicina61020269)
Supplement: Supplementary file 1 [file medicina-61-00269-s001.zip › medicina-3416753-supplementary.pdf]

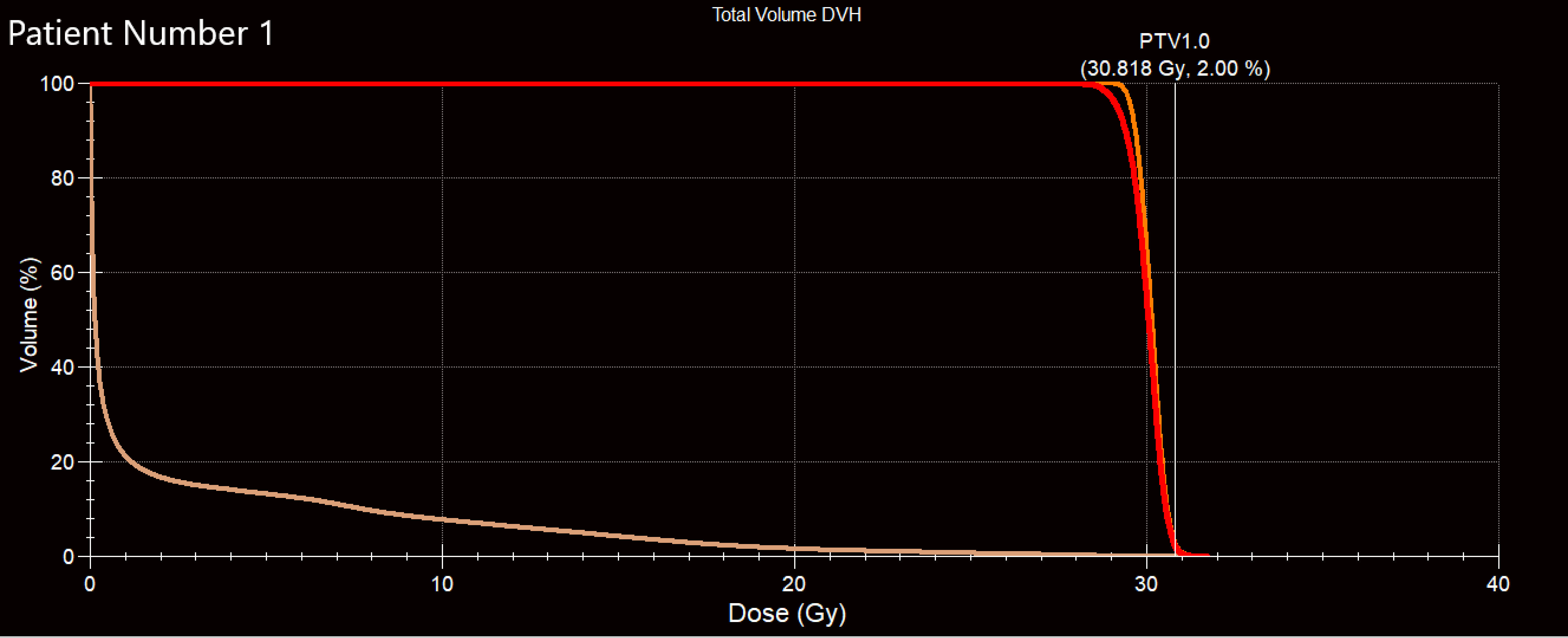

DVH Statistics

Dosimetric Criteria Statistics Display

	Structure	Volume (cm³)	Min. Dose (Gy)	Max. Dose (Gy)	Mean Dose (Gy)	Ref. Vol. (cm³)	Ref. Vol. (%)	Ref. Dose (Gy)	D..	% in ...	Is in SS	Heterogeneity Index	Conformity Index
	PTV1.0	682.398	27.530	31.711	30.000	13.648	2.00	30.818		100.00	yes	1.07	0.95
						668.750	98.00	28.873					
	patient(Unsp.Tiss.)	38458.557	0.001	30.721	1.984					100.00	no	886.99	
	CTV1.0	297.162	28.720	31.711	30.145					100.00	yes	1.04	

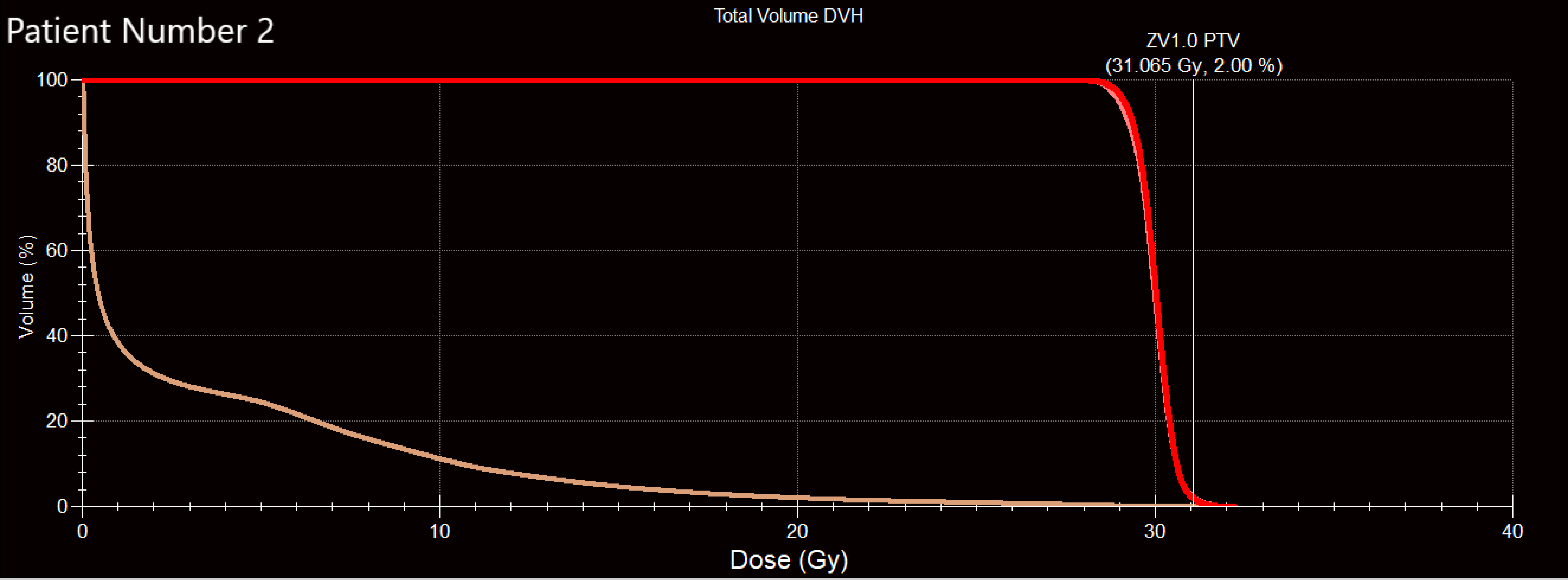

DVH Statistics

Dosimetric Criteria | Statistics | Display

	Structure	Volume (cm³)	Min. Dose (Gy)	Max. Dose (Gy)	Mean Dose (Gy)	Ref. Vol. (cm³)	Ref. Vol. (%)	Ref. Dose (Gy)	D	% in...	Is in SS	Heterogeneity Index	Conformity Index
	ZV1.0 PTV	506.493	26.847	32.207	30.000	10.130	2.00	31.065		100.00	yes	1.07	0.93
						496.363	98.00	28.842					
	patient(Unsp.Tiss.)	23579.586	0.016	30.149	3.168					100.00	no	282.72	
	ZV1.0 CTV	220.293	26.847	32.207	29.956					100.00	yes	1.06	

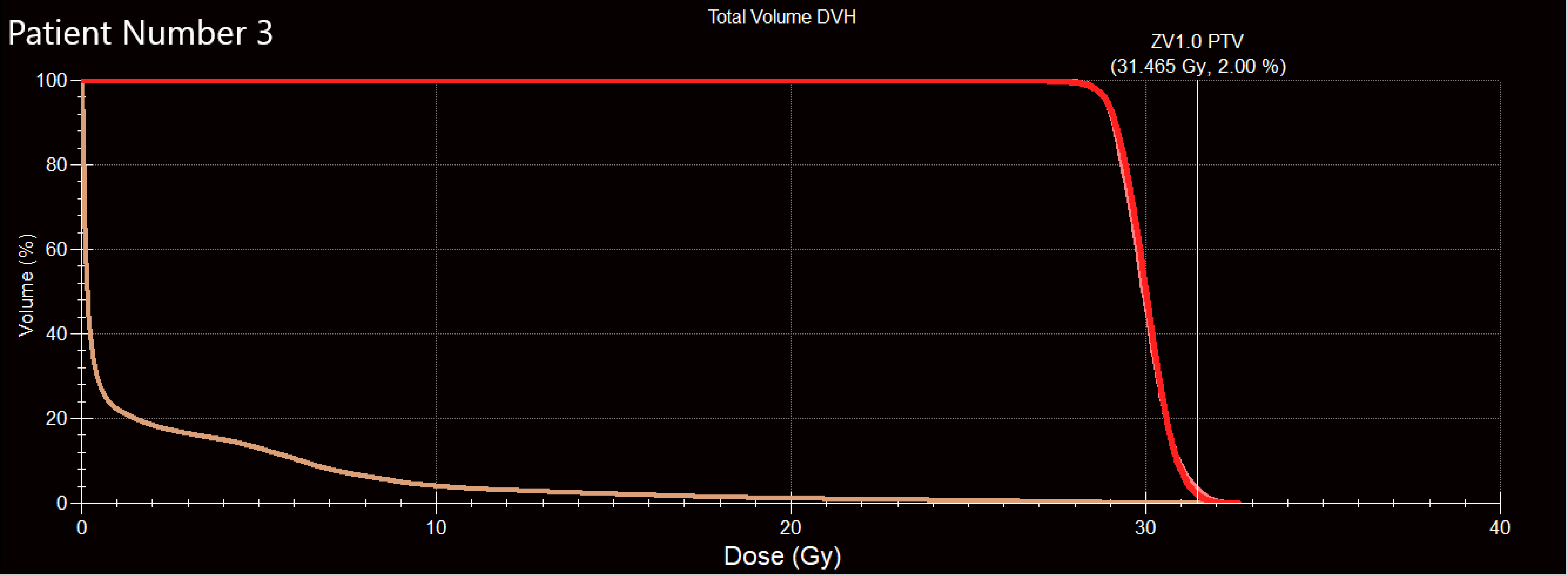

DVH Statistics

Dosimetric Criteria | Statistics | Display

	Structure	Volume (cm³)	Min. Dose (Gy)	Max. Dose (Gy)	Mean Dose (Gy)	Ref. Vol. (cm³)	Ref. Vol. (%)	Ref. Dose (Gy)	Dosi…	% in …	Is i…	Heterogeneity Index	Conformity Index
	ZV1.0 PTV	264.519	23.051	32.594	30.000	5.290	2.00	31.465		100.00	yes	1.09	0.88
						259.229	98.00	28.560					
	patient(Unsp.Tiss.)	14333.058	0.012	31.603	1.696					100.00	no	219.93	
	ZV1.0 CTV	87.642	26.752	32.383	29.974					100.00	yes	1.08	

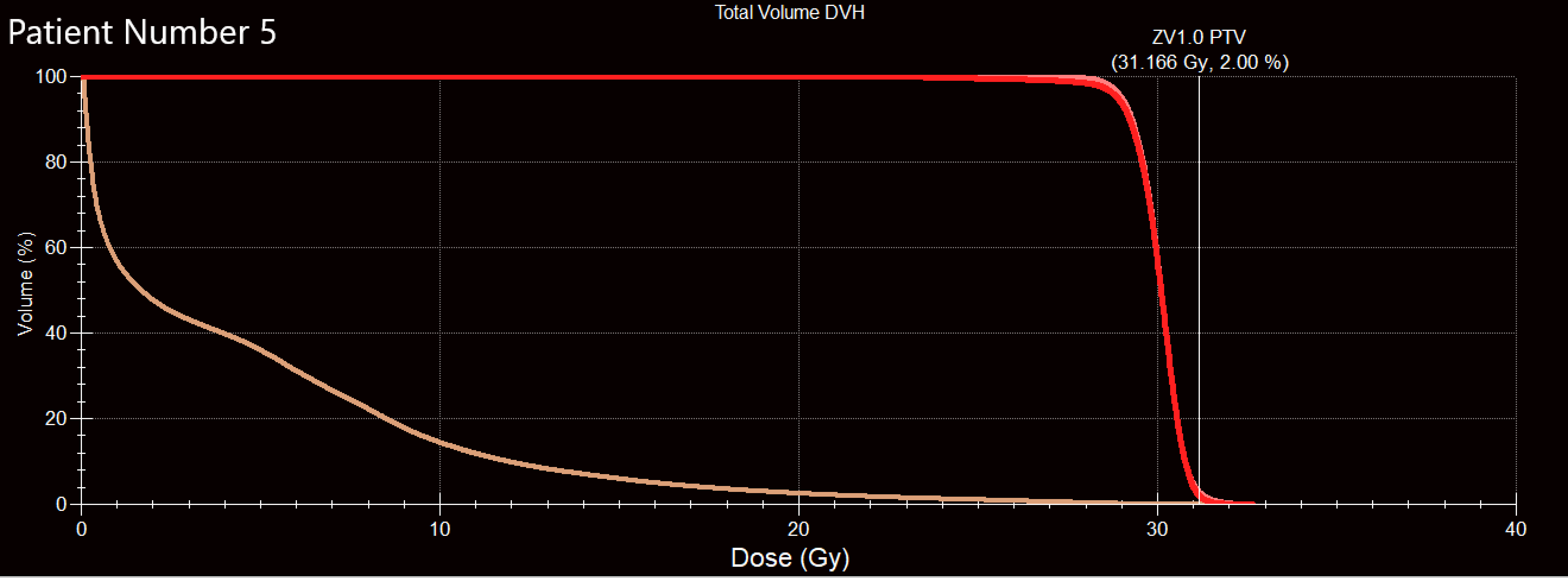

DVH Statistics

Dosimetric Criteria | Statistics | Display

	Structure	Volume (cm³)	Min. Dose (Gy)	Max. Dose (Gy)	Mean Dose (Gy)	Ref. Vol. (cm³)	Ref. Vol. (%)	Ref. Dose (Gy)		D...	% in...	Is in SS	Heterogeneity Index	Conformity Index
	ZV1.0 PTV	751.140	21.737	32.394	30.000	15.023	2.00	31.166			100.00	yes	1.08	0.93
						736.117	98.00	28.308						
	patient(Unsp.Tiss.)	23715.342	0.033	31.392	4.482						100.00	no	138.79	
	ZV1.0 CTV	350.001	25.915	32.632	30.074						100.00	yes	1.07	

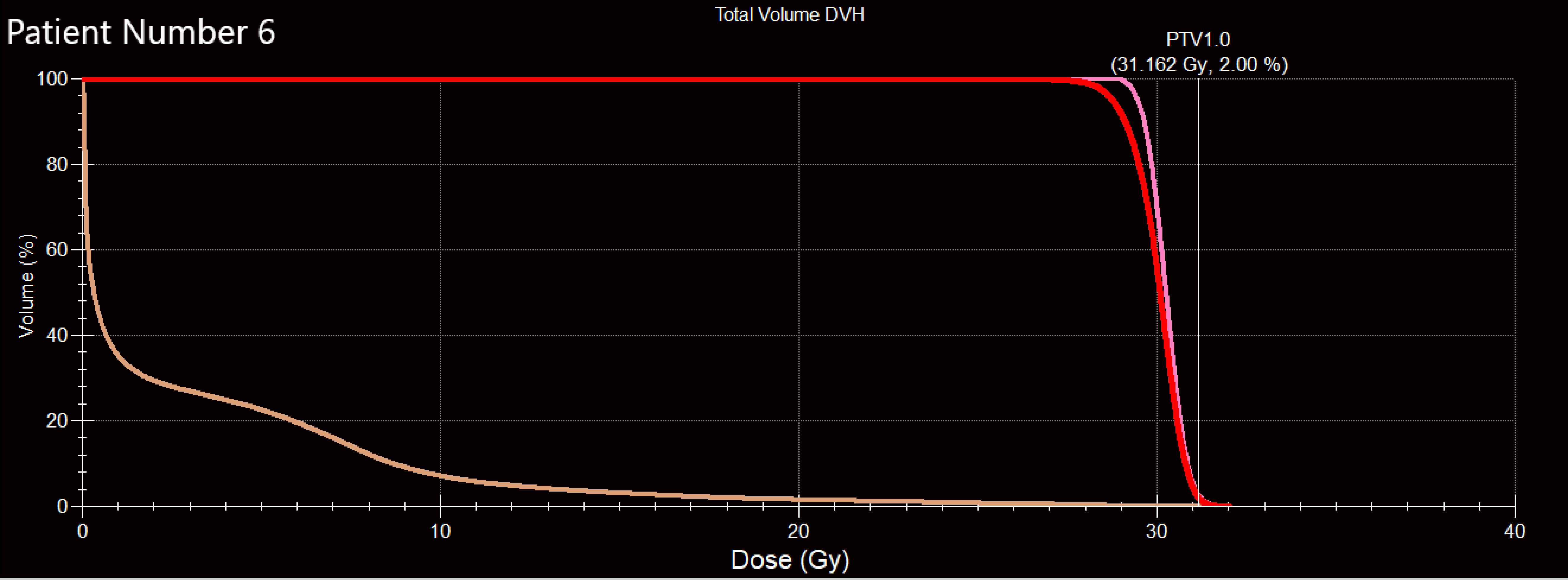

DVH Statistics

Dosimetric Criteria | Statistics | Display

	Structure	Volume (cm³)	Min. Dose (Gy)	Max. Dose (Gy)	Mean Dose (Gy)	Ref. Vol. (cm³)	Ref. Vol. (%)	Ref. Dose (Gy)		Do...	% i...	Is in SS	Heterogeneity Index	Conformity Index
	PTV1.0	318.438	25.072	32.006	30.000	6.369	2.00	31.162			100.00	yes	1.08	0.94
						312.069	98.00	28.355						
	patient(Unsp.Tiss.)	10986.705	0.017	29.968	2.712						100.00	no	241.90	
	CTV1.0	111.753	28.834	32.006	30.255						100.00	yes	1.05	

Patient Number 7

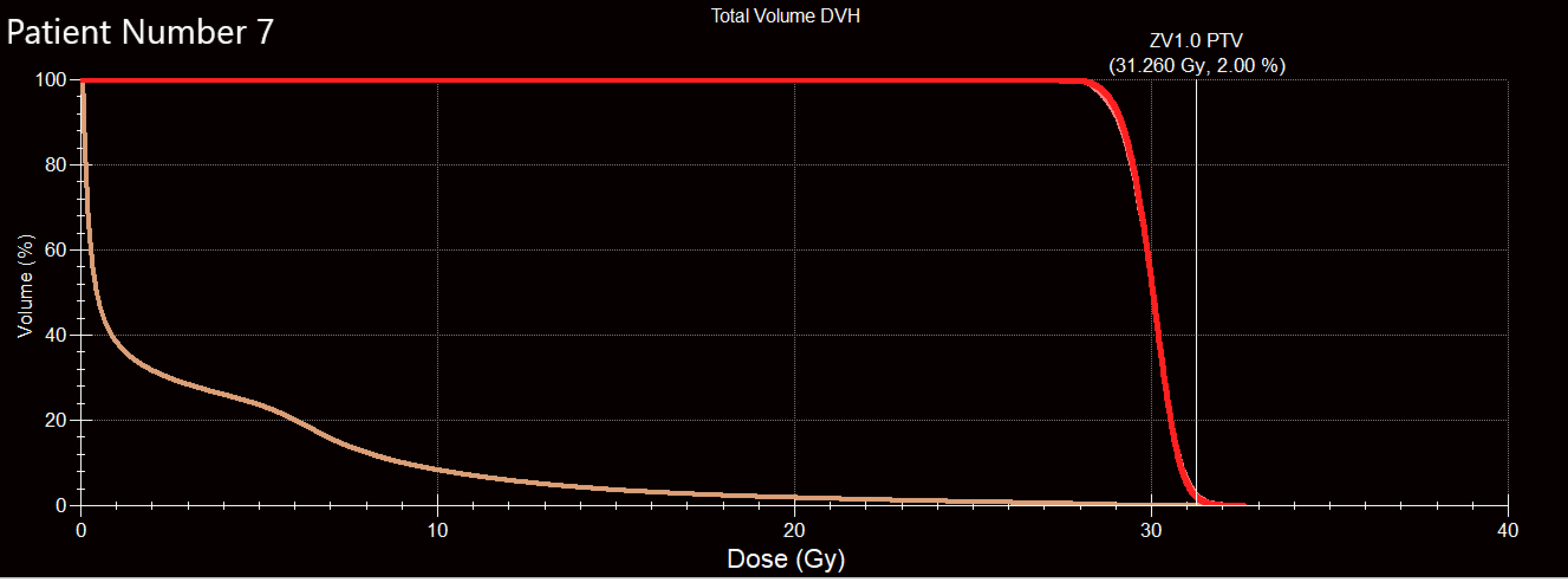

DVH Statistics

Dosimetric Criteria | Statistics | Display

	Structure	Volume (cm³)	Min. Dose (Gy)	Max. Dose (Gy)	Mean Dose (Gy)	Ref. Vol. (cm³)	Ref. Vol. (%)	Ref. Dose (Gy)		D..	% in Volume	Is...	Heterogeneity Index	Conformity Index
■	ZV1.0 PTV	474.633	26.665	32.579	30.000	9.493	2.00	31.260			100.00	yes	1.08	0.88
						465.140	98.00	28.562						
■	patient(Unsp.Tiss.)	26355.888	0.030	30.739	2.956						100.00	no	156.11	
■	ZV1.0 CTV	206.793	26.941	32.578	29.990						100.00	yes	1.08	
